# Active monitoring vs. spontaneous reporting of antineoplastic drug–related adverse drug reactions: evidence from the Chinese hospital pharmacovigilance system

**DOI:** 10.3389/frhs.2025.1741402

**Published:** 2026-01-22

**Authors:** Hao Jing, Du Jie, Wang Zhong, Ma Xiao, Zhang Qingxuan, Li Sha, Zhang Shuai, Xiao Yunyan, Lv Mingxiao, Liu Yahui

**Affiliations:** Department of Pharmacy, Xingtai People’s Hospital, Xingtai, Hebei, China

**Keywords:** active monitoring, adverse drug reactions, antineoplastic agents, Chinese hospital pharmacovigilance system (CHPS), pharmacovigilance, signal detection, spontaneous reporting

## Abstract

**Background:**

Adverse drug reactions (ADRs) remain a major barrier to safe and effective cancer therapy. Existing pharmacovigilance systems predominantly rely on spontaneous reporting, which suffers from underreporting and delays. The Chinese Hospital Pharmacovigilance System (CHPS) provides an opportunity for active monitoring using multidimensional hospital data.

**Methods:**

We conducted a retrospective cohort study, including 500 patients who received chemotherapy, targeted therapy, or immunotherapy. ADRs were identified through CHPS, classified by the Common Terminology Criteria for Adverse Events (CTCAE), and assessed using both active monitoring and spontaneous reporting. Signal detection employed disproportionality analyses (PRR, ROR, IC). Risk factors were analyzed with logistic regression, and predictive models for severe ADRs were evaluated with ROC curve analysis.

**Results:**

The overall ADR incidence was 37.0% (185/500), with 28.1% classified as severe. Hematologic (29.7%), gastrointestinal (26.0%), and skin/mucosal (19.5%) events were most common. Severe ADRs led to hospitalization (34.6%), treatment discontinuation (23.1%), and death (9.6%). Independent risk factors included age ≥65 years, polypharmacy, hepatic/renal dysfunction, and prolonged drug exposure (≥14 days). Signal detection confirmed known associations and identified potential novel signals, including skin hyperpigmentation with PD-1/PD-L1 inhibitors and cardiotoxicity with tyrosine kinase inhibitors. Active monitoring detected more ADRs than spontaneous reporting (160 vs. 50, *P* < 0.001) and provided earlier detection (mean 4.2 vs. 10.7 days). Predictive modeling demonstrated strong performance of the multivariable model (AUC = 0.82), with active monitoring outperforming spontaneous reporting (AUC = 0.84 vs. 0.72).

**Conclusion:**

CHPS-based active monitoring improves the detection, timeliness, and predictive assessment of ADRs compared with spontaneous reporting. These findings support the integration of active monitoring into hospital pharmacovigilance systems and highlight novel safety signals requiring further validation.

## Introduction

Adverse drug reactions (ADRs) represent a major global health concern, as they are a leading cause of hospital admissions, readmissions, prolonged hospital stays, and increased mortality (1–4). The effective monitoring and management of ADRs are therefore central to ensuring drug safety and protecting public health (5, 6). Pharmacovigilance systems have been established worldwide to support the systematic detection, reporting, and evaluation of ADRs (7–9).

Despite these advances, most existing systems still rely heavily on spontaneous reporting (10). While widely implemented, this passive approach has well-recognized limitations (2, 7, 11). Underreporting, delayed reporting, duplicate entries, and incomplete clinical information are common challenges that undermine data quality (8, 9). Moreover, spontaneous reporting systems are typically biased toward severe or rare events, resulting in under-recognition of mild and moderate ADRs that also have clinical and economic implications (5, 12, 13). These limitations make it difficult to conduct high-quality signal detection, post-marketing safety evaluation, and timely regulatory intervention, thus highlighting the urgent need for more proactive and data-driven approaches to pharmacovigilance (8–10, 14).

The Chinese Hospital Pharmacovigilance System (CHPS), developed by the National Center for ADRs Monitoring, is an informatics-based dataset and integration platform designed to support sentinel hospitals in detecting and reporting ADRs and adverse events (15). It also facilitates targeted monitoring, post-marketing drug safety evaluation, and the acquisition of pharmacovigilance information for research and regulatory purposes (15). Furthermore, the system provides valuable evidence to support hospital pharmacy practice, clinical pharmacology, and public health policy-making (15–18).

Although CHPS has been gradually implemented in sentinel hospitals across China, systematic research based on this platform remains limited. In particular, comparative studies between active monitoring and spontaneous reporting are scarce, and the clinical value of signal detection and the identification of novel ADRs has not been fully validated. These gaps restrict the broader application of CHPS in pharmacovigilance research and clinical decision-making. Therefore, we conducted a retrospective, single-center study using data from the CHPS to explore the occurrence and characteristics of antineoplastic drug–related ADRs, to compare CHPS-based active monitoring with conventional spontaneous reporting, and to apply disproportionality analyses and multivariable modeling to generate safety signals and risk prediction hypotheses. Rather than providing definitive causal evidence or fully validated prediction tools, this study is intended as an exploratory, hypothesis-generating evaluation of how real-world hospital data can be leveraged to enhance pharmacovigilance in oncology. The findings are meant to inform future multi-center and externally validated studies, as well as the further development of hospital-based active surveillance platforms.

## Methods

### Study design and setting

This retrospective cohort study was conducted at Xingtai People's Hospital, a tertiary general hospital in Hebei Province, China, using routinely collected data from the CHPS. The study period covered all consecutive adult patients who received at least one course of systemic antineoplastic therapy between January 2020 and December 2024 and met the predefined inclusion and exclusion criteria. As a single-center analysis based on one institutional CHPS implementation, the study was designed primarily as a hypothesis-generating evaluation of active monitoring vs. spontaneous reporting and not as a definitive multi-center validation of pharmacovigilance performance.

No random sampling or convenience sampling was conducted; the final sample of 500 patients reflects the entire eligible population during the study period. This approach minimizes selection bias and ensures that the cohort is representative of real-world oncology practice at our institution.

The study protocol was reviewed and approved by the Ethics Committee of Xingtai People's Hospital, and all procedures were carried out in accordance with the Declaration of Helsinki and relevant national regulations on biomedical research ethics.

### Inclusion and exclusion criteria

Patients were eligible for inclusion if they met the following criteria: (1) Age ≥18 years at the time of diagnosis or treatment; (2) Received at least one course of antineoplastic therapy, including chemotherapy, targeted therapy, or immune checkpoint inhibitors; (3) Had complete electronic medical records and medication records available in the Chinese Hospital Pharmacovigilance System (CHPS).

Patients were excluded if they met any of the following conditions: (1) Incomplete or severely missing clinical information, including missing baseline demographics, treatment details, or outcome data; (2) Use of non-oncologic medications as the primary therapy, without clear documentation of antineoplastic drug exposure; (3) Lack of follow-up information, making it impossible to assess ADR occurrence, severity, or clinical outcomes.

### Data sources

All data were obtained from the Chinese Hospital Pharmacovigilance System (CHPS), which integrates multi-dimensional information from electronic health records. The dataset included:

#### Patient demographics and baseline characteristics

Patient demographics and baseline characteristics included sex, age at the initiation of treatment, and body mass index (BMI). Lifestyle-related information, such as smoking and alcohol consumption history (where available), was also collected. In addition, patients’ medical history and comorbid conditions were recorded, including hypertension, diabetes mellitus, cardiovascular disease, and chronic hepatic or renal disorders.

#### Clinical information

Clinical information encompassed the primary cancer diagnosis, histological subtype, and tumor stage at baseline. Details of prior and concomitant therapies were collected, including chemotherapy, targeted therapy, immunotherapy, radiotherapy, and supportive medications. In addition, treatment cycles, cumulative drug exposure, and any dose modifications or adjustments during the course of therapy were systematically documented.

#### Laboratory and imaging examinations

Laboratory and imaging data were retrieved from CHPS to support the evaluation of treatment safety. Hematological indices included complete blood counts, such as white blood cell, neutrophil, hemoglobin, and platelet levels. Hepatic and renal function tests comprised alanine aminotransferase (ALT), aspartate aminotransferase (AST), serum creatinine, and estimated glomerular filtration rate (eGFR). Cardiac assessments, including electrocardiography (ECG) and echocardiography, were collected to monitor left ventricular ejection fraction and QT interval. In addition, imaging examinations such as computed tomography (CT), magnetic resonance imaging (MRI), or ultrasound were reviewed when relevant to suspected adverse drug reaction (ADR) events.

#### Adverse drug reaction (ADR) data

ADR-related information was systematically extracted from CHPS. Event characteristics included the date and time of onset as well as the latency from the initiation of drug therapy. The type of ADR was classified according to the system organ class (SOC) framework. Severity was graded in accordance with the Common Terminology Criteria for Adverse Events. Clinical outcomes were also documented, including complete recovery, persistence with sequelae, hospitalization, treatment discontinuation, or death.

### Definition and classification of ADRs

Adverse drug reactions (ADRs) were defined according to the criteria of the World Health Organization (WHO) and the National Adverse Drug Reaction Monitoring Center (NADRMC) of China. Severe ADRs were classified as events of grade 3 or higher based on the Common Terminology Criteria for Adverse Events (CTCAE), or those resulting in hospitalization or death. ADRs were categorized by system organ class (SOC), including but not limited to hematologic disorders, gastrointestinal events, skin and mucosal reactions, immune-related toxicities, and hepatic or renal impairment. Clinical outcomes were graded as complete recovery, survival with sequelae, or death.

All suspected ADR events identified through CHPS triggers or clinical documentation were independently reviewed by two trained clinical pharmacists who applied standardized CTCAE criteria to assign severity grades. Any discrepancies were adjudicated by a senior oncologist to ensure consistency. To evaluate inter-rater reliability, a random 20% subsample of ADR cases was reassessed, yielding a Cohen's *κ* coefficient of 0.82, indicating strong agreement. This multi-reviewer process was implemented to minimize subjective variability and enhance the robustness of ADR classification.

### Monitoring approaches

Two monitoring approaches were applied in this study. Active monitoring was conducted through the CHPS platform, which automatically captured abnormal prescription records, laboratory test results, and imaging findings. These data were further reviewed and confirmed by clinical physicians, enabling near real-time identification and early warning of ADRs. In contrast, spontaneous reporting relied on voluntary submissions by healthcare providers or patients. Reports were typically made only for severe events, which resulted in well-recognized issues such as underreporting and delayed reporting, particularly for mild and moderate ADRs.

### Data completeness and missing-data handling

To evaluate variable-level completeness within CHPS before analysis. Demographic and medication-exposure variables had <2% missingness, whereas selected laboratory parameters (ALT, AST, eGFR) showed 5%–12% missingness. Based on the observed patterns and clinical workflow, missingness was considered most compatible with a missing-at-random (MAR) mechanism. Variables with <5% missingness were analyzed using complete-case analysis. For variables with ≥5% missingness, multiple imputation by chained equations (*m* = 5) was performed, incorporating demographics, comorbidities, treatment regimen, and ADR status as predictors. Results from the imputed datasets were pooled using Rubin's rules, and sensitivity analyses confirmed consistency with complete-case findings.

### Misclassification minimization procedures

Given that CHPS is based on routinely collected clinical data, several procedures were implemented to reduce potential ADR misclassification. All candidate ADR events—identified via abnormal laboratory values, medication changes, or clinician-entered notes—were independently reviewed by two trained clinical pharmacists using standardized CTCAE criteria, with disagreements resolved by a senior oncologist. Event timestamps were cross-checked against medication administration records to verify temporal plausibility. ADR categories were assigned according to a predefined SOC-based taxonomy to minimize subjective variation. Despite these safeguards, some degree of misclassification remains possible due to variability in routine clinical documentation and the inherent limitations of retrospective EHR extraction.

### Statistical analysis

All statistical analyses were performed using SPSS software and R software. A two-sided *P* value of <0.05 was considered statistically significant. Missing data were handled by either multiple imputation or listwise deletion, depending on the extent and distribution of missingness. Signal detection was performed using three widely adopted disproportionality methods in pharmacovigilance: the proportional reporting ratio (PRR), the reporting odds ratio (ROR), and the information component (IC). A signal was considered positive if the predefined thresholds were met, namely PRR ≥ 2 with *χ*² ≥ 4, lower limit of the 95% confidence interval (CI) for ROR > 1, or lower limit of the 95% CI for IC > 0.

Potential risk factors associated with ADRs were first assessed by univariate analysis using the chi-square test for categorical variables and the Student's *t* test for continuous variables. Variables showing statistical significance in univariate analysis were subsequently entered into a multivariate logistic regression model. Covariates included age, sex, body mass index (BMI), comorbidities, number of concomitant medications, and hepatic/renal function status. Results were reported as odds ratios (ORs) with 95% confidence intervals (CIs).

A predictive model for severe ADRs was developed using multivariate logistic regression. Model performance was evaluated by calculating the area under the receiver operating characteristic (ROC) curve (AUC), as well as sensitivity, specificity, and the Youden index at the optimal cut-off point. Model comparisons were conducted using the DeLong test to assess differences in AUC. To address potential overfitting from using the same dataset for model development and evaluation, we conducted bootstrap internal validation with 1,000 resamples, generating optimism-corrected AUC estimates. Calibration was assessed using calibration plots comparing predicted vs. observed probabilities and the Hosmer–Lemeshow goodness-of-fit test. In addition to the multivariable model, comparative analyses were performed for (1) single-factor models (e.g., age, number of concomitant drugs), (2) the multivariable combined model, and (3) models based on active monitoring vs. spontaneous reporting.

## Results

### Baseline characteristics of the study population

A total of 500 patients were enrolled in this study, including 280 males (56.4%) and 220 females (43.6%). The overall mean age was 58.7 ± 9.6 years, with males being slightly older than females (60.1 ± 9.4 vs. 56.8 ± 9.7 years). Regarding cancer type, lung cancer was the most frequent diagnosis (31.7%), followed by breast cancer (23.5%), gastric cancer (18.5%), and other tumors (26.2%). As expected, breast cancer occurred almost exclusively in females (54.0%), whereas lung cancer (39.8%) and gastric cancer (24.7%) were more common among males. With respect to treatment regimens, chemotherapy was the predominant modality (40.4%), followed by targeted therapy (28.2%), immunotherapy (19.6%), and combination regimens (12.4%). Chemotherapy and targeted therapy were more frequently administered in male patients, while immunotherapy and combination therapies were relatively more common among females. Detailed baseline characteristics are presented in Table 1.

**Table 1:** Baseline characteristics of the study population.

Variable	Overall (*N* = 500)	Male (*n* = 280)	Female (*n* = 220)
Sample size (*N*)	500	280	220
Male, *n* (%)	280 (56.42)	280 (100.00)	0 (0.00)
Female, *n* (%)	220 (43.65)	0 (0.00)	220 (100.00)
Mean age (years, mean ± SD)	58.73 ± 9.58	60.05 ± 9.36	56.77 ± 9.72
Lung cancer, *n* (%)	158 (31.65)	112 (39.82)	47 (21.96)
Breast cancer, *n* (%)	118 (23.54)	0 (0.00)	121 (54.02)
Gastric cancer, *n* (%)	92 (18.48)	68 (24.66)	21 (9.28)
Other tumors, *n* (%)	133 (26.21)	98 (35.52)	32 (14.05)
Chemotherapy, *n* (%)	202 (40.37)	121 (43.09)	79 (35.97)
Targeted therapy, *n* (%)	139 (28.16)	88 (31.67)	51 (23.09)
Immunotherapy, *n* (%)	97 (19.58)	52 (18.09)	49 (22.41)
Combination regimen, *n* (%)	63 (12.43)	22 (7.63)	39 (17.95)

Data are presented as numbers (percentages) unless otherwise indicated. SD, standard deviation.

### Overall incidence of ADRs

**Figure 1:**
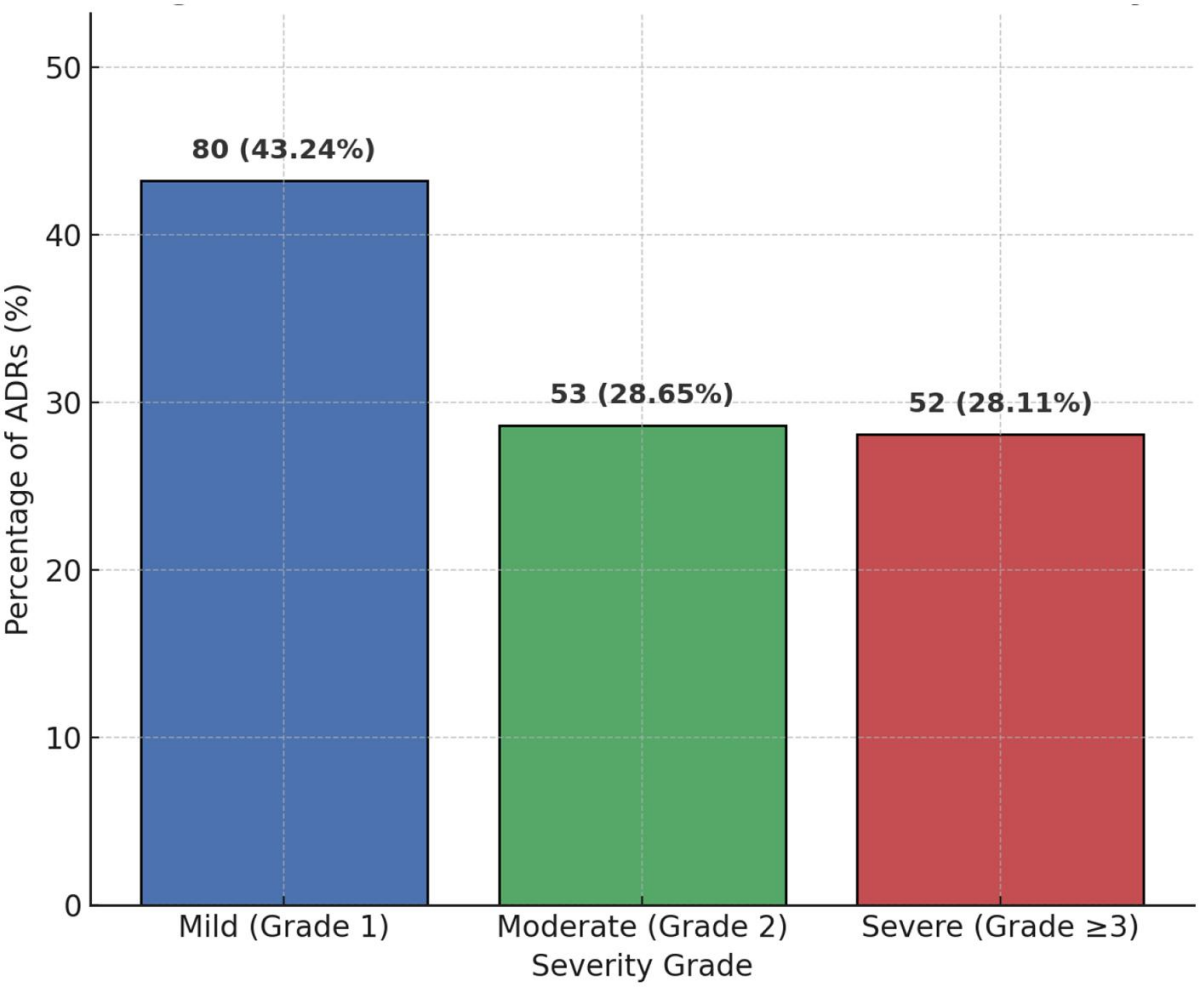
Distribution of ADR incidence and severity. The bar chart illustrates the proportion of adverse drug reactions (ADRs) stratified by severity grade (CTCAE). Among the 185 identified ADRs, 80 (43.24%) were mild (Grade 1), 53 (28.65%) were moderate (Grade 2), and 52 (28.11%) were severe (Grade ≥ 3). Data are presented as number of cases with corresponding percentages.

Among the 500 patients included in the analysis, a total of 185 adverse drug reactions (ADRs) were identified, corresponding to an overall incidence of 37.00% (185/500). Of these, 52 cases (28.11%) were classified as severe ADRs (CTCAE grade ≥ 3). When comparing different surveillance strategies, active monitoring captured 160 cases, whereas spontaneous reporting identified only 50 cases during the same period. The distribution of ADR incidence and severity levels is illustrated in Figure 1.

### Types and clinical manifestations of ADRs

Among the 185 ADR cases, hematologic disorders were the most frequently reported (29.73%), predominantly presenting as leukopenia, anemia, and thrombocytopenia. Gastrointestinal reactions ranked second (25.95%), including nausea, vomiting, diarrhea, and mucositis. Skin and mucosal reactions accounted for 19.46%, with rash, pruritus, and stomatitis being the most common presentations.

**Figure 2:**
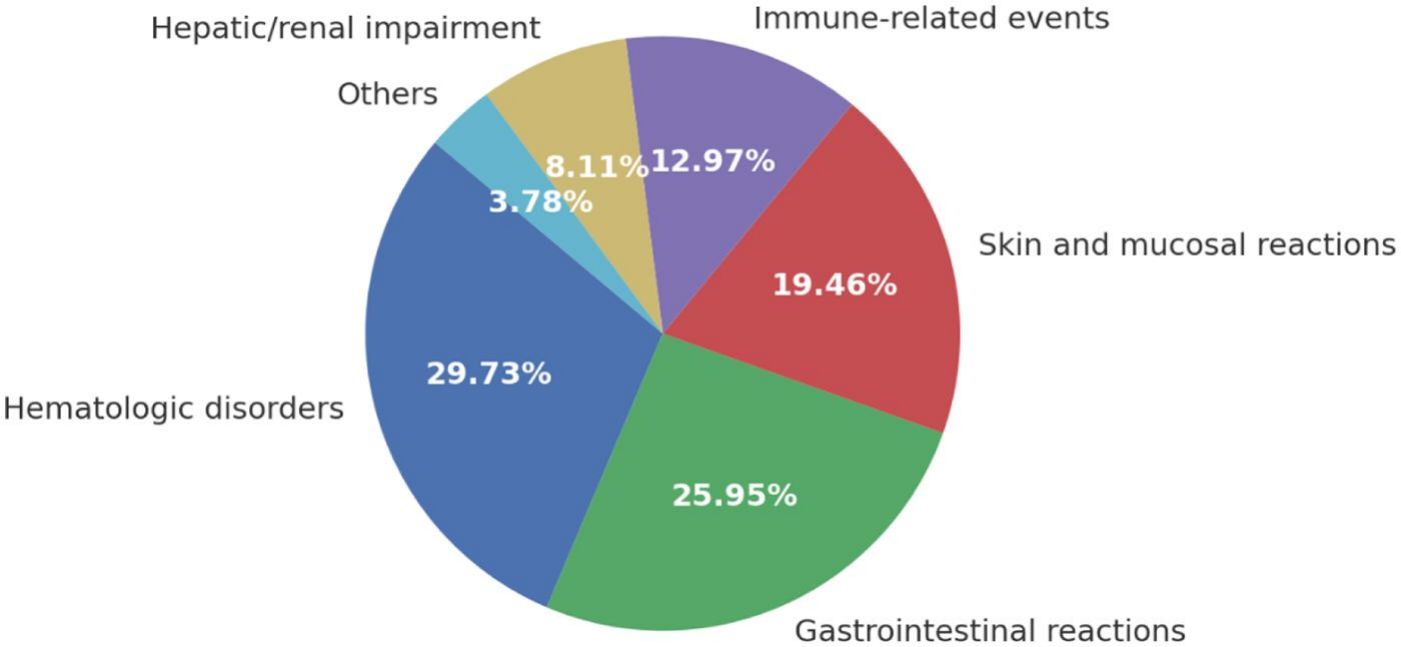
Distribution of ADR types. The pie chart illustrates the relative proportions of different adverse drug reaction (ADR) categories identified in the study cohort (*N* = 185). Hematologic disorders were the most frequent (29.73%), followed by gastrointestinal reactions (25.95%), skin and mucosal reactions (19.46%), immune-related events (12.97%), hepatic/renal impairment (8.11%), and others (3.78%). Percentages are shown as proportions of all ADR cases.

Immune-related events represented 12.97% of all ADRs and were primarily associated with PD-1/PD-L1 inhibitors, manifesting as thyroid dysfunction, hepatitis, and pneumonitis. Hepatic and renal impairments were observed in 8.11% of cases, most commonly drug-induced hepatotoxicity and transient renal function decline. Other less frequent manifestations (3.78%) included infusion-related reactions and cardiovascular adverse events. The proportional distribution of ADR categories is depicted in Figure 2.

### Serious ADRs and clinical outcomes

Among the 185 reported ADRs, 52 cases (28.11%) were classified as severe (CTCAE grade ≥ 3). Of these, 18 patients (34.62%) required hospitalization, 12 patients (23.08%) discontinued treatment, and 5 patients (9.62%) died due to ADR-related complications. Regarding clinical outcomes, 31 patients (59.62%) fully recovered after appropriate management, 16 patients (30.77%) improved but remained with residual symptoms, and 5 patients (9.62%) ultimately died. Detailed outcomes of severe ADRs are presented in Table 2.

**Table 2:** Clinical outcomes of severe ADRs.

Clinical outcome	Cases (*n*)	Percentage (%)
Hospitalization due to ADR	18	34.62
Treatment discontinuation	12	23.08
Death	5	9.62
Full recovery	31	59.62
Improvement with sequelae	16	30.77
Death (repeated for outcome status)	5	9.62

Data are presented as numbers and percentages. Some patients experienced multiple outcomes (e.g., hospitalization followed by recovery or death).

### Risk factors analysis

Univariate analysis identified several factors significantly associated with ADRs, including age ≥65 years, female sex, concomitant use of ≥3 medications, hepatic/renal dysfunction, BMI ≥25 kg/m², history of cardiovascular disease, and drug exposure ≥14 days (all *P* < 0.05). Multivariate logistic regression confirmed that age ≥65 years (OR = 1.84, 95% CI: 1.13–2.99, *P* = 0.015), concomitant use of ≥3 medications (OR = 2.27, 95% CI: 1.39–3.71, *P* = 0.001), hepatic/renal dysfunction (OR = 1.90, 95% CI: 1.07–3.38, *P* = 0.028), and drug exposure ≥14 days (OR = 1.76, 95% CI: 1.05–2.94, *P* = 0.031) were independent risk factors for ADRs. Female sex, BMI ≥25 kg/m², and cardiovascular comorbidity showed increased risk in univariate analysis but were not statistically significant after adjustment (Table 3). Detailed estimates for both univariate analyses and the expanded multivariate model are provided in Supplementary Table S1.

**Table 3:** Univariate and multivariate logistic regression analysis of risk factors for ADRs.

Variable	Univariate OR (95% CI)	*P* value	Multivariate OR (95% CI)	*P* value
Age ≥65 years	1.78 (1.11–2.87)	0.018	1.84 (1.13–2.99)	0.015
Female sex	1.39 (1.00–1.94)	0.049	1.32 (0.94–1.87)	0.098
Concomitant use of ≥3 drugs	2.46 (1.53–3.95)	<0.001	2.27 (1.39–3.71)	0.001
Hepatic/renal dysfunction	2.01 (1.17–3.45)	0.011	1.90 (1.07–3.38)	0.028
BMI ≥25 kg/m²	1.41 (1.02–1.96)	0.039	1.28 (0.90–1.83)	0.161
Cardiovascular disease	1.55 (1.07–2.26)	0.021	1.31 (0.87–1.96)	0.193
Duration of exposure ≥14 days	1.81 (1.11–2.95)	0.017	1.76 (1.05–2.94)	0.031

OR, odds ratio; CI, confidence interval.

### Signal detection and findings

**Figure 3:**
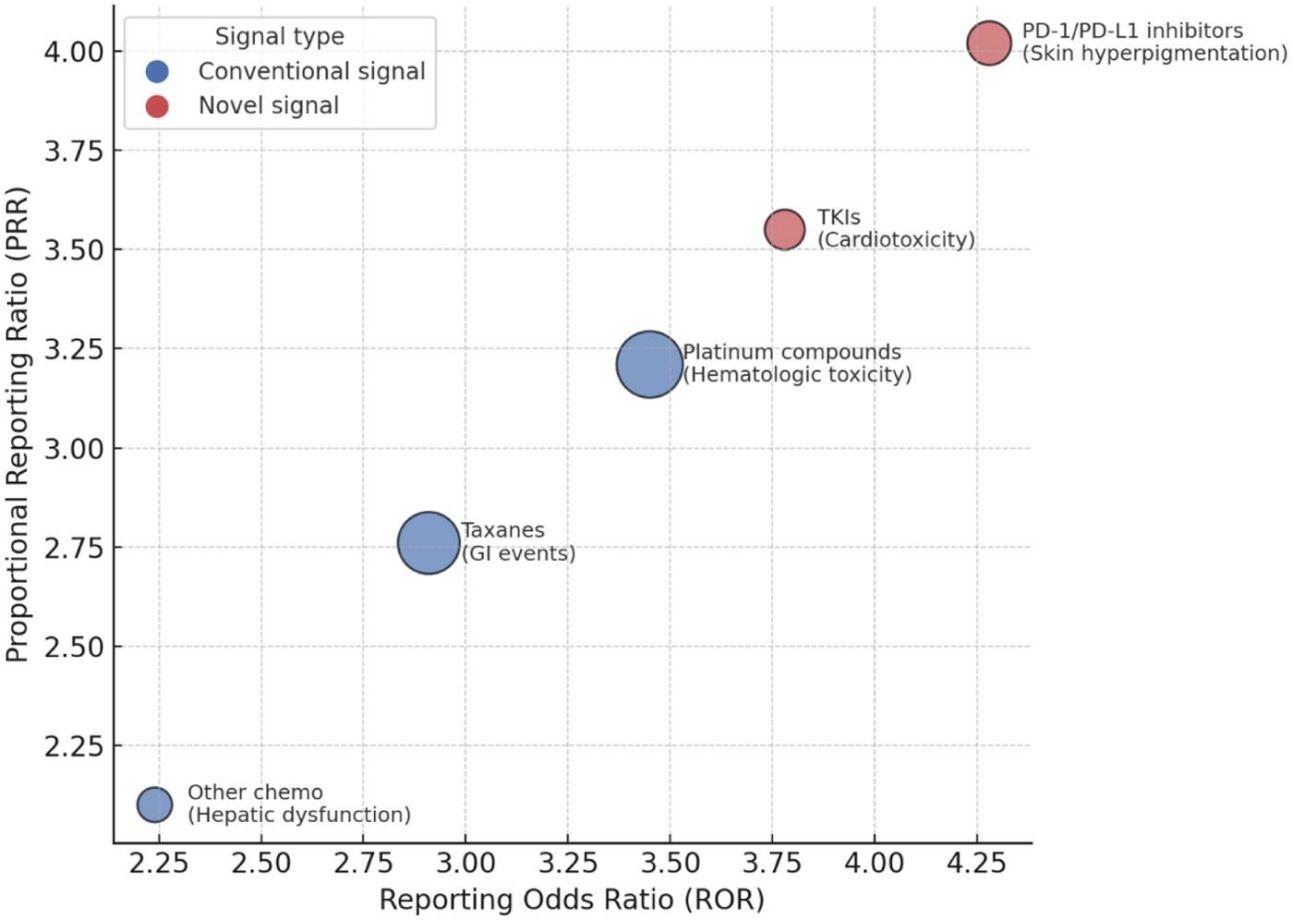
Drug–ADR signal strength bubble plot. The bubble plot depicts the strength of drug–ADR associations identified by disproportionality analysis. The *x*-axis represents the reporting odds ratio (ROR), and the *y*-axis represents the proportional reporting ratio (PRR). Bubble size corresponds to the number of reported cases. Blue bubbles indicate conventional signals consistent with known drug labels, whereas red bubbles highlight potential novel signals, including skin hyperpigmentation associated with PD-1/PD-L1 inhibitors and cardiotoxicity related to tyrosine kinase inhibitors (TKIs).

Disproportionality analyses were performed using proportional reporting ratio (PRR), reporting odds ratio (ROR), and information component (IC). Several conventional signals, consistent with current drug labeling, were detected, including hematologic toxicities associated with platinum compounds and gastrointestinal adverse events associated with taxanes. PD-1/PD-L1 inhibitors demonstrated a potential association with skin hyperpigmentation, which has not been comprehensively documented in existing product information. In addition, tyrosine kinase inhibitors (TKIs) showed a signal for cardiotoxicity, specifically QT prolongation and arrhythmia, suggesting a need for enhanced monitoring in clinical practice. The detailed results of signal detection are presented in Table 4, and the relative strength of drug–ADR associations is illustrated in Figure 3.

**Table 4:** Signal detection results of drug-ADR pairs.

Drug class/agent	ADR type	PRR	ROR (95% CI)	IC (95% CI)	Signal status
Platinum compounds	Hematologic toxicity	3.21	3.45 (2.11–5.64)	1.25 (0.88–1.62)	Conventional
Taxanes	Gastrointestinal events	2.76	2.91 (1.84–4.62)	1.11 (0.73–1.48)	Conventional
PD-1/PD-L1 inhibitors	Skin hyperpigmentation	4.02	4.28 (2.33–7.84)	1.62 (1.12–2.01)	Novel
TKIs	Cardiotoxicity (QT prolongation/arrhythmia)	3.55	3.78 (2.05–6.94)	1.45 (1.03–1.87)	Novel
Other chemotherapeutics	Hepatic dysfunction	2.10	2.24 (1.21–4.12)	0.92 (0.55–1.29)	Conventional

PRR, proportional reporting ratio; ROR, reporting odds ratio; IC, information component.

### Active monitoring vs. spontaneous reporting

A comparative analysis demonstrated that active monitoring substantially outperformed spontaneous reporting in detecting ADRs. Active monitoring identified 160 cases, whereas spontaneous reporting captured only 50 cases during the same observation period. Moreover, active monitoring achieved a wider coverage of ADR types, including mild and moderate events that were frequently underreported in spontaneous systems.

**Figure 4:**
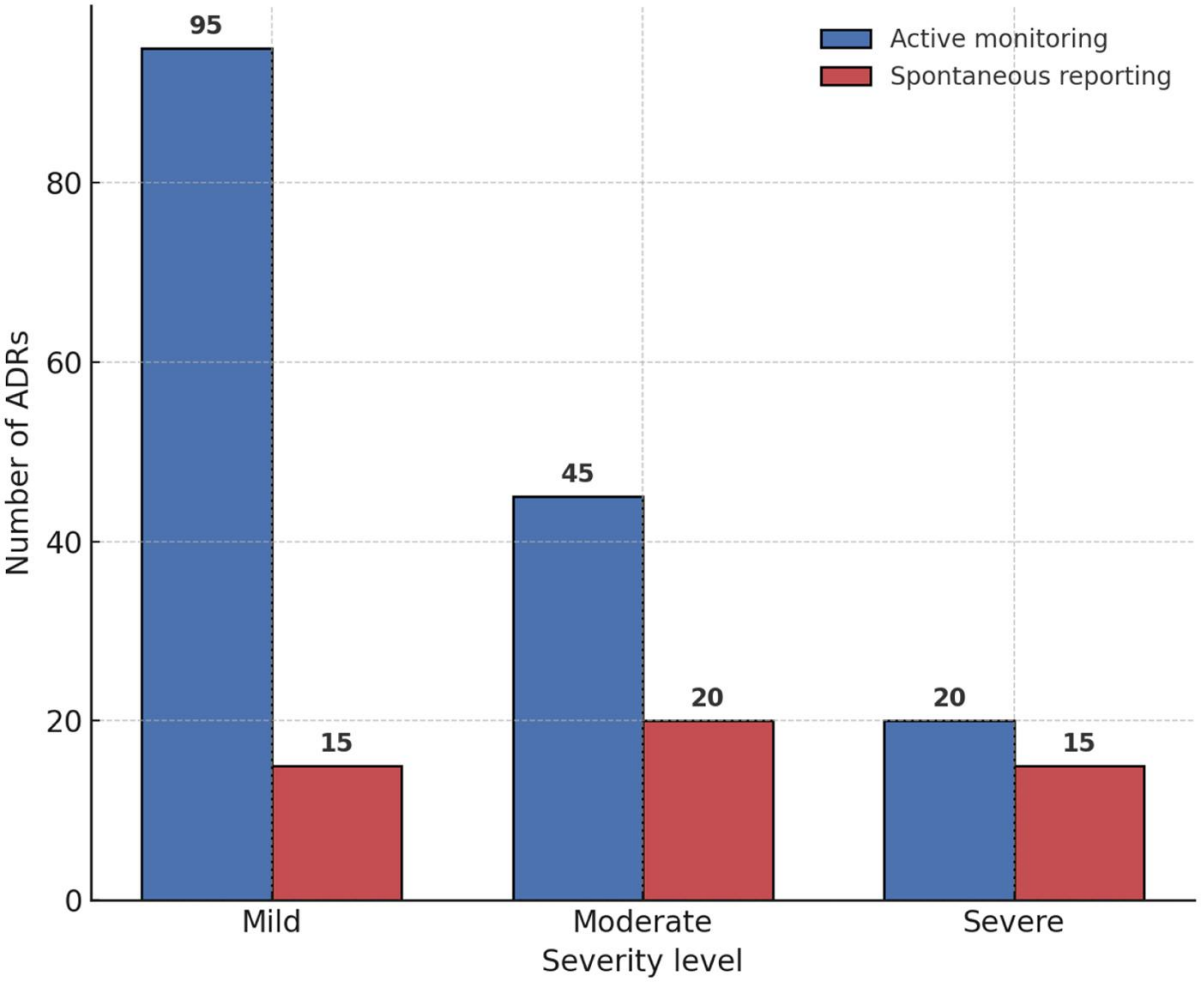
Drug–ADR signal strength bubble plot. The bubble plot depicts the disproportionality analysis of drug–ADR associations. The *x*-axis shows the reporting odds ratio (ROR), and the *y*-axis represents the proportional reporting ratio (PRR). Bubble size corresponds to the number of reports, with blue bubbles representing conventional signals and red bubbles highlighting novel signals (skin hyperpigmentation with PD-1/PD-L1 inhibitors and cardiotoxicity with TKIs).

On average, active monitoring detected ADRs 6.5 days earlier than spontaneous reporting, highlighting its practical value for timely pharmacovigilance and clinical intervention. Detailed comparisons are presented in Table 5, and the differences in ADR detection between the two methods are illustrated in Figure 4.

**Table 5:** Comparison of active monitoring and spontaneous reporting.

Parameter	Active monitoring	Spontaneous reporting	*P* value
Number of ADRs detected	160	50	<0.001
Mild ADRs, *n* (%)	95 (59.38)	15 (30.00)	<0.001
Moderate ADRs, *n* (%)	45 (28.12)	20 (40.00)	0.041
Severe ADRs, *n* (%)	20 (12.50)	15 (30.00)	0.007
Mean time to detection (days)	4.2 ± 1.6	10.7 ± 2.4	<0.001

ADR, adverse drug reaction.

### Predictive performance with ROC analysis

To evaluate the predictive value for severe ADRs, receiver operating characteristic (ROC) curve analyses were performed. The multivariable logistic regression model demonstrated good discriminatory ability, with an AUC of 0.82 (95% CI: 0.75–0.88). At the optimal cut-off, the sensitivity and specificity were 78.0% and 74.5%, respectively. After bootstrap internal validation (1,000 iterations), the optimism-corrected AUCs were 0.82 and 0.80, respectively, indicating limited overfitting. Calibration analysis demonstrated good agreement between predicted and observed risks. The calibration plot showed no systematic deviation across risk strata, and the Hosmer–Lemeshow test was non-significant (*P* = 0.41), indicating acceptable model fit. Calibration slope, intercept, Brier score, and optimism-corrected AUC metrics are summarized in Supplementary Table S2.

**Figure 5:**
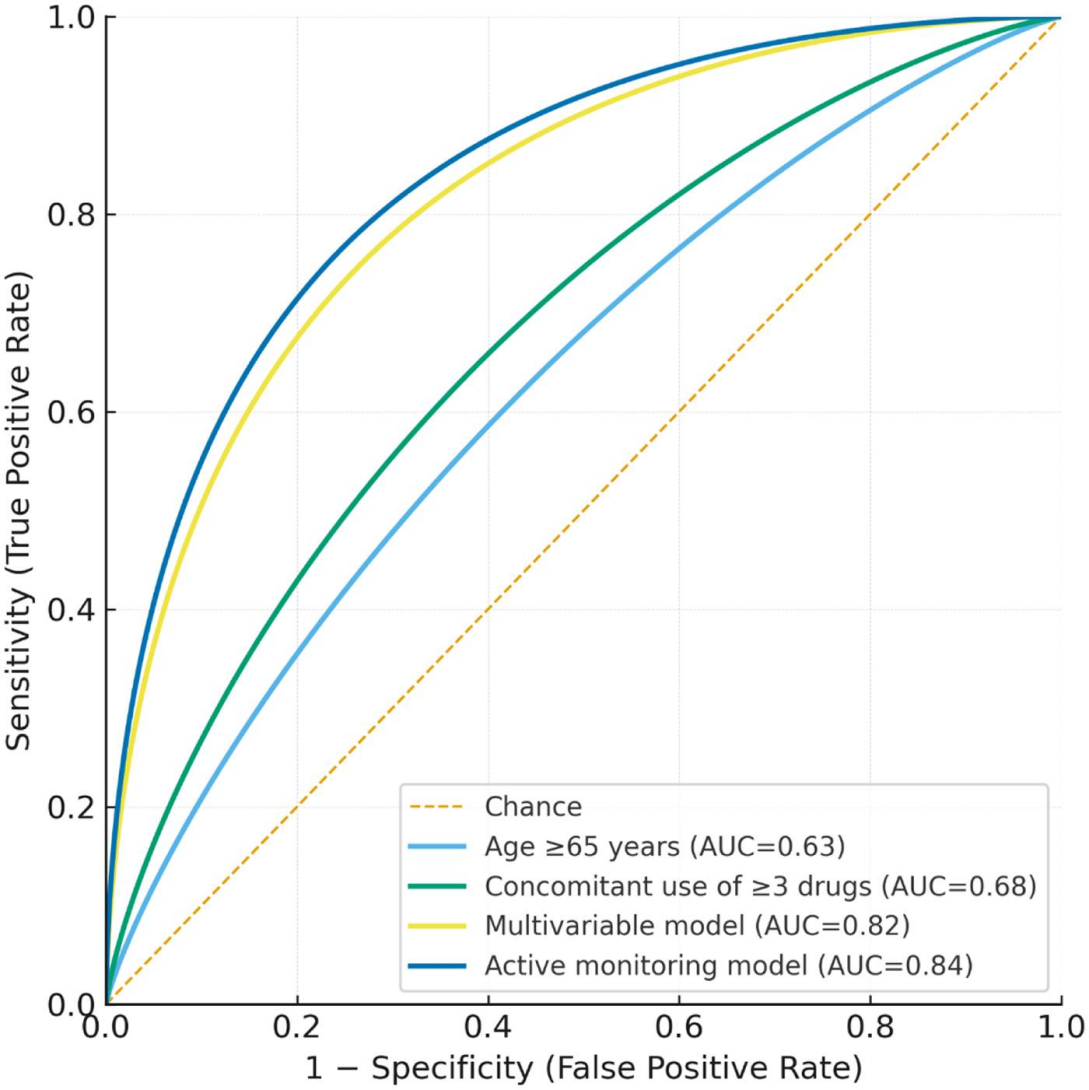
ROC curves of predictive models. The ROC curves illustrate the predictive performance of different models for severe ADRs. Curve 1 represents age ≥65 years, curve 2 represents concomitant use of ≥3 drugs, curve 3 represents the multivariable model, and curve 4 compares active monitoring with spontaneous reporting.

Compared with single-variable models (age ≥65 years: AUC = 0.63; concomitant use of ≥3 drugs: AUC = 0.68), the multivariable model showed significantly improved predictive performance (*P* < 0.05, DeLong test). Furthermore, the active monitoring–based model (AUC = 0.84) outperformed the spontaneous reporting model (AUC = 0.72), underscoring the added value of proactive pharmacovigilance. Detailed model performance metrics are provided in Table 6, and ROC curves are shown in Figure 5.

**Table 6:** Predictive performance of different models for severe ADRs.

Model	AUC (95% CI)	Cut-off	Sensitivity (%)	Specificity (%)	Youden index
Age ≥65 years	0.63 (0.55–0.71)	0.40	62.5	60.2	0.23
Concomitant use of ≥3 drugs	0.68 (0.60–0.75)	0.45	66.7	63.0	0.30
Multivariable model	0.82 (0.75–0.88)	0.52	78.0	74.5	0.53
Active monitoring model	0.84 (0.77–0.89)	0.50	80.5	76.3	0.57
Spontaneous reporting model	0.72 (0.65–0.80)	0.48	70.2	67.5	0.38

AUC, area under the curve.

### Missing data and model robustness

Variable-level missingness and the full specification of the MICE imputation model, including predictors used for chained equations, are summarized in Supplementary Table S3. To evaluate the influence of additional confounder adjustment, a direct comparison between the original and expanded multivariate logistic regression models is presented in Supplementary Table S4, showing minimal changes in effect estimates and preserved significance for key predictors.

## Discussion

In this retrospective analysis based on the CHPS, several important findings were observed. The overall incidence of ADRs was considerable, with hematologic, gastrointestinal, and skin/mucosal events most frequently observed. A proportion of patients developed severe ADRs, leading to hospitalization, treatment discontinuation, or death. Risk factor analysis highlighted older age, polypharmacy, and hepatic or renal dysfunction as significant contributors. Signal detection confirmed established associations and suggested novel signals, including skin hyperpigmentation with immune checkpoint inhibitors and cardiotoxicity with tyrosine kinase inhibitors. Active monitoring proved superior to spontaneous reporting in both detection rate and timeliness. Finally, predictive modeling showed that multivariable models performed better than single-variable models, and that active monitoring–based models outperformed those based on spontaneous reporting.

The overall incidence of ADRs observed in this study was broadly comparable with international pharmacovigilance databases, though the distribution of event types showed some variation. According to reports from WHO VigiBase, FDA FAERS, and EMA EudraVigilance, hematologic and gastrointestinal toxicities remain the most frequently reported categories for antineoplastic drugs, consistent with our findings (19–31). However, our study identified a relatively higher proportion of skin and mucosal reactions, which may reflect differences in population characteristics, prescribing practices, and reporting systems between China and Western countries. This discrepancy is likely due to the use of the CHPS platform, which enables active monitoring and captures mild and moderate ADRs that are often underreported in passive systems. These findings underscore the added value of integrating real-world hospital data into pharmacovigilance, allowing for a more comprehensive assessment of ADR burden in oncology patients.

The distribution of ADR types in our cohort aligns with the prevailing literature on antineoplastic toxicities (32–35). Consistent with prior studies, chemotherapy was predominantly associated with hematologic events (e.g., neutropenia, anemia, thrombocytopenia) and gastrointestinal reactions (nausea, vomiting, diarrhea), whereas immune checkpoint inhibitors most frequently produced immune-related toxicities (thyroid dysfunction, hepatitis, pneumonitis) (36–38). Targeted therapies showed a mixed profile, including dermatologic/mucosal reactions and hepatic/renal laboratory abnormalities, which is also consistent with published reports (39, 40). Minor deviations in proportions (e.g., a relatively higher share of skin/mucosal events) may reflect differences in case mix, supportive-care protocols, and the use of active surveillance (CHPS), which is more sensitive to capturing mild and moderate events than passive systems.

The proportion of severe ADRs in our study was comparable to international reports in oncology populations. The main consequences included hospitalization, treatment discontinuation, and occasional deaths. Mortality was low but clinically relevant, aligning with global estimates that severe ADRs remain an important cause of morbidity and, rarely, mortality. Signal detection confirmed known associations such as hematologic toxicity with platinum agents and gastrointestinal events with taxanes.

The disproportionality analysis reproduced several established associations, such as hematologic toxicities with platinum agents and gastrointestinal AEs with taxanes. A few additional associations—including skin hyperpigmentation with PD-1/PD-L1 inhibitors and cardiotoxicity with tyrosine kinase inhibitors—showed elevated PRR, ROR, and IC values. However, these patterns must be interpreted with caution. The modest sample size and single-center nature of the dataset limit the stability of disproportionality estimates and increase the possibility of spurious findings. Reporting bias, differences in clinician documentation practices, and confounding by indication or treatment selection may further influence these associations. Demographic or practice characteristics specific to our hospital population may also contribute. Accordingly, these findings should be regarded as exploratory, hypothesis-generating safety signals, requiring confirmation in larger multi-center datasets or external pharmacovigilance systems such as FAERS or VigiBase.

In interpreting our findings, several considerations warrant a more cautious perspective. First, the disproportionality patterns identified in this study should be viewed as exploratory, hypothesis-generating signals, rather than evidence of novel ADRs, as confirmation in larger multi-center datasets or external pharmacovigilance systems (e.g., FAERS, VigiBase) is required. Second, although CHPS-based active monitoring detected more ADRs than spontaneous reporting, this difference likely reflects the underutilization of spontaneous reporting at our institution and should not be interpreted as inherent superiority of the active-surveillance framework. Instead, the findings illustrate how automated monitoring can complement—not replace—traditional reporting practices. Third, compared with established active-surveillance platforms such as the FDA Sentinel Initiative or EHR-linked pharmacovigilance programs in Europe, CHPS operates on a smaller scale but shares core features, including structured EHR integration and automated event identification.

Several mechanisms may help explain the observed patterns of ADRs and associated risk factors. Older patients are more vulnerable due to age-related changes in pharmacokinetics and pharmacodynamics, combined with the higher prevalence of comorbidities (31). Polypharmacy increases the likelihood of pharmacokinetic and pharmacodynamic drug–drug interactions, thereby raising the risk of adverse outcomes (41, 42). Similarly, baseline hepatic or renal impairment can reduce drug clearance, leading to drug accumulation and enhanced toxicity. The mechanisms underlying different ADR types are consistent with the pharmacology of the respective therapies. Cytotoxic chemotherapy commonly causes bone marrow suppression, resulting in hematologic toxicities. Immune checkpoint inhibitors can trigger aberrant immune activation, giving rise to immune-related adverse events such as dermatitis, thyroiditis, and hepatitis (43, 44). TKIs may disrupt ion-channel function, predisposing patients to cardiac toxicities, including QT prolongation and arrhythmias (41, 45). Differences between monitoring approaches may also reflect these mechanistic considerations. Active monitoring systems are capable of detecting a broader spectrum of ADRs, including mild and moderate events that may be clinically silent or underreported, whereas spontaneous reporting tends to capture predominantly severe events that prompt clinical recognition and reporting.

This study has several important limitations. First, it was conducted at a single tertiary hospital, and all analyses were based on one institutional implementation of CHPS. As such, the case-mix, prescribing patterns, and reporting culture may differ from those in other settings, which limits the generalizability of our findings. Second, the retrospective design and reliance on routinely collected CHPS data raise the possibility of incomplete capture, misclassification of ADRs, and residual confounding, despite our efforts to standardize ADR ascertainment and grading. Third, the multivariable models and ROC analyses were developed and evaluated within the same dataset, and no external or independent validation cohort was available. The reported AUCs should therefore be interpreted as optimistic and hypothesis-generating rather than definitive performance estimates. Finally, the disproportionality analyses were based on a moderate sample size and were not complemented by external pharmacovigilance databases; consequently, the observed safety signals, particularly those considered “potentially novel,” require confirmation in larger, multi-center and externally validated studies. Future studies should focus on multicenter, large-scale prospective cohorts to validate these findings, assess the clinical relevance of newly detected signals, and refine predictive models for ADR risk stratification.

## Conclusion

In conclusion, this study utilized the CHPS to systematically evaluate the incidence, characteristics, and risk factors of antineoplastic drug–related ADRs, while also applying signal detection and predictive modeling. The findings demonstrate that active monitoring outperforms spontaneous reporting in both scope and timeliness, and highlight potential novel signals that merit further validation. These results provide evidence to support safer clinical use of antineoplastic agents and to inform the optimization of hospital-based pharmacovigilance systems in China.

## Data Availability

The original contributions presented in the study are included in the article/Supplementary Material, further inquiries can be directed to the corresponding author.
