# Supplementary material for: Active monitoring vs. spontaneous reporting of antineoplastic drug–related adverse drug reactions: evidence from the Chinese hospital pharmacovigilance system
Source: Front Health Serv. 2026 Jan 22;5:1741402. doi: 10.3389/frhs.2025.1741402 (PMC12872567; doi:10.3389/frhs.2025.1741402)
Supplement: Supplementary file 1 [file Table1.docx]

**Supplementary Table S1. Univariate and Expanded Multivariate Logistic Regression Analysis of Risk Factors for ADRs**

**Table S1. Logistic Regression Models for ADR Risk (Univariate, Base Multivariate, Expanded Multivariate)**

| **Variable** | **Univariate OR (95% CI)** | **P value** | **Multivariate Model A OR (95% CI)** | **P value** | **Expanded Multivariate Model B OR (95% CI)** | **P value** |
| --- | --- | --- | --- | --- | --- | --- |
| **Age ≥65 years** | 1.78 (1.11–2.87) | 0.018 | 1.84 (1.13–2.99) | 0.015 | **1.79 (1.09–2.93)** | **0.021** |
| **Female sex** | 1.39 (1.00–1.94) | 0.049 | 1.32 (0.94–1.87) | 0.098 | 1.28 (0.90–1.82) | 0.144 |
| **Concomitant use of ≥3 drugs** | 2.46 (1.53–3.95) | <0.001 | 2.27 (1.39–3.71) | 0.001 | **2.19 (1.33–3.60)** | **0.002** |
| **Hepatic/renal dysfunction** | 2.01 (1.17–3.45) | 0.011 | 1.90 (1.07–3.38) | 0.028 | **1.88 (1.05–3.34)** | **0.032** |
| **BMI ≥25 kg/m²** | 1.41 (1.02–1.96) | 0.039 | 1.28 (0.90–1.83) | 0.161 | 1.22 (0.86–1.75) | 0.253 |
| **Cardiovascular disease** | 1.55 (1.07–2.26) | 0.021 | 1.31 (0.87–1.96) | 0.193 | 1.22 (0.81–1.84) | 0.333 |
| **Treatment exposure ≥14 days** | 1.81 (1.11–2.95) | 0.017 | 1.76 (1.05–2.94) | 0.031 | **1.71 (1.02–2.88)** | **0.041** |
| **Tumor type (solid vs. hematologic)** | — | — | — | — | 1.12 (0.79–1.59) | 0.519 |
| **Advanced stage** | — | — | — | — | 1.19 (0.83–1.71) | 0.348 |
| **Treatment modality (chemo / targeted / immunotherapy)** | — | — | — | — | 1.08 (0.73–1.61) | 0.702 |
| **ECOG ≥2** | — | — | — | — | 1.29 (0.82–2.02) | 0.269 |
| **Comorbidities (HTN, DM, CKD)** | — | — | — | — | 1.17 (0.80–1.70) | 0.409 |

**Notes:**

- Model A = original multivariate model.
- Model B = expanded model adjusting for tumor type, disease stage, treatment modality, ECOG performance status, comorbidities, and baseline organ function.
- OR = odds ratio; CI = confidence interval; HTN = hypertension; DM = diabetes mellitus; CKD = chronic kidney disease.
- Bold values indicate statistically significant predictors in the expanded model.

**Supplementary Table S2. ROC Performance and Internal Validation of Predictive Models**

**Table S2. ROC Statistics, Optimism-Corrected AUC, and Calibration Metrics (Bootstrap = 1,000 iterations)**

| **Metric** | **Active-Monitoring Model** | **Combined Clinical–Laboratory Model** |
| --- | --- | --- |
| **Apparent AUC** | 0.84 | 0.82 |
| **Optimism (Bootstrap 1,000 resamples)** | 0.020 | 0.018 |
| **Optimism-Corrected AUC** | **0.82** | **0.80** |
| **95% CI (bootstrap percentile)** | 0.78–0.86 | 0.76–0.84 |
| **Sensitivity (Youden index)** | 0.77 | 0.74 |
| **Specificity (Youden index)** | 0.71 | 0.68 |
| **Calibration slope** | 0.94 | 0.92 |
| **Calibration intercept** | 0.03 | 0.05 |
| **Hosmer–Lemeshow P value** | 0.41 | 0.38 |
| **Brier score** | 0.152 | 0.167 |

**Notes:**

- Apparent AUC = performance in original dataset.
- Optimism-corrected AUC = internal validation estimate.
- Calibration slope near 1 indicates good calibration; Brier score <0.20 indicates acceptable predictive accuracy.
- No evidence of overfitting was observed.

**Supplementary Table S3. Missing-Data Summary and Variables Included in MICE Imputation**

**Table S3. Missingness Pattern and Multiple Imputation Specification**

| **Variable** | **Type** | **Missingness (%)** | **Included in MICE Model** | **Notes** |
| --- | --- | --- | --- | --- |
| **Age** | Continuous | 0.0 | Yes | Complete data |
| **Sex** | Categorical | 0.0 | Yes | — |
| **BMI** | Continuous | 1.3 | Yes | <5%; complete-case also consistent |
| **Comorbidities (HTN/DM/CVD)** | Categorical | 0.8 | Yes | — |
| **Tumor type** | Categorical | 0.0 | Yes | — |
| **Disease stage** | Categorical | 0.0 | Yes | — |
| **Treatment modality** | Categorical | 0.0 | Yes | — |
| **ALT** | Continuous | 11.2 | Yes | MICE applied; MAR assumption justified |
| **AST** | Continuous | 10.4 | Yes | — |
| **eGFR** | Continuous | 8.7 | Yes | — |
| **Hemoglobin** | Continuous | 4.6 | Yes | Near threshold; imputed for consistency |
| **Neutrophil count** | Continuous | 5.3 | Yes | ≥5%; MICE |
| **Medication exposure duration** | Continuous | 0.0 | Yes | — |
| **Number of concomitant drugs** | Continuous | 0.0 | Yes | — |
| **ADR status** | Binary outcome | 0.0 | Yes | Included as auxiliary variable |

**MICE Imputation Model Specification**

- **Imputation method:** Multiple Imputation by Chained Equations (MICE)
- **Number of imputations:** m = 5 datasets
- **Predictors included in imputation model：**
  Age, sex, BMI, comorbidities, tumor type, disease stage, treatment modality, eGFR, ALT, AST, hemoglobin, neutrophil count, exposure duration, number of concomitant drugs, and ADR outcome
- **Assumed missing-data mechanism:** Missing At Random (MAR), justified based on workflow patterns
- **Pooling method:** Rubin’s rules
- **Sensitivity analysis:** Complete-case vs. imputed datasets produced consistent effect estimates

**Supplementary Table S4. Comparison of Original and Expanded Multivariate Logistic Regression Models for ADR Prediction**

**Table S4. Side-by-Side Comparison of Multivariate Model A (Original) and Model B (Expanded Confounder Adjustment)**

| **Variable** | **Model A: Adjusted OR (95% CI)** | **P value** | **Model B: Expanded Adjusted OR (95% CI)** | **P value** | **Change in Estimate (%)** | **Interpretation** |
| --- | --- | --- | --- | --- | --- | --- |
| **Age ≥65 years** | 1.84 (1.13–2.99) | 0.015 | **1.79 (1.09–2.93)** | **0.021** | –2.7% | Stable independent risk factor |
| **Female sex** | 1.32 (0.94–1.87) | 0.098 | 1.28 (0.90–1.82) | 0.144 | –3.0% | Association attenuated; confounded |
| **Concomitant use of ≥3 drugs** | 2.27 (1.39–3.71) | 0.001 | **2.19 (1.33–3.60)** | **0.002** | –3.5% | Robust independent predictor |
| **Hepatic/renal dysfunction** | 1.90 (1.07–3.38) | 0.028 | **1.88 (1.05–3.34)** | **0.032** | –1.1% | Minimal change; effect stable |
| **BMI ≥25 kg/m²** | 1.28 (0.90–1.83) | 0.161 | 1.22 (0.86–1.75) | 0.253 | –4.7% | Non-significant; weakened further |
| **Cardiovascular disease** | 1.31 (0.87–1.96) | 0.193 | 1.22 (0.81–1.84) | 0.333 | –6.9% | Effect further attenuated |
| **Duration of exposure ≥14 days** | 1.76 (1.05–2.94) | 0.031 | **1.71 (1.02–2.88)** | **0.041** | –2.8% | Stable independent predictor |
| **Tumor type (solid vs hematologic)** | — | — | 1.12 (0.79–1.59) | 0.519 | — | Not a meaningful confounder |
| **Advanced stage** | — | — | 1.19 (0.83–1.71) | 0.348 | — | No independent effect |
| **Treatment modality** | — | — | 1.08 (0.73–1.61) | 0.702 | — | Not associated after adjustment |
| **ECOG ≥2** | — | — | 1.29 (0.82–2.02) | 0.269 | — | Not independently predictive |
| **Comorbidities (HTN, DM, CKD)** | — | — | 1.17 (0.80–1.70) | 0.409 | — | No effect in adjusted model |
