# Supplementary material for: Active monitoring vs. spontaneous reporting of antineoplastic drug–related adverse drug reactions: evidence from the Chinese hospital pharmacovigilance system
Source: Front Health Serv. 2026 Jan 22;5:1741402. doi: 10.3389/frhs.2025.1741402 (PMC12872567; doi:10.3389/frhs.2025.1741402)
Supplement: Supplementary file 2 [file Datasheet1.pdf]

R Script: CHPS_ADR Analysis

```
# ==== ■■■■■■■■■■■■CHPS-ADR■ ==== # ■■■ pkgs <-
c("readxl","dplyr","broom","pROC","ggplot2") invisible(lapply(setdiff(pkgs,
rownames(installed.packages())), install.packages)) lapply(pkgs, library,
character.only = TRUE) # 1) ■■■■
--------------------------------------------------------------- data_path <-
"CHPS_ADR_RawData.xlsx" df <- readxl::read_excel(data_path) # 2) ■■■■■
------------------------------------------------------------- df <- df %>% mutate(
Sex = factor(Sex), Cancer_Type = factor(Cancer_Type), Therapy_Type =
factor(Therapy_Type), Hepatic_Renal_Dysfunction = factor(Hepatic_Renal_Dysfunction,
levels=c(0,1)), `Duration_≥14d` = factor(`Duration_≥14d`, levels=c(0,1)), ADR =
factor(ADR, levels=c(0,1)), ADR_Severity = factor(ADR_Severity,
levels=c("None","Mild","Moderate","Severe")), Monitoring_Type =
factor(Monitoring_Type, levels=c("None","Active","Spontaneous")), Age_Group =
factor(Age_Group, levels=c(0,1), labels=c("<65","≥65")), BMI25 = factor(ifelse(BMI
>= 25, 1, 0), levels=c(0,1), labels=c("<25","≥25")), Drugs3 =
factor(ifelse(Concomitant_Drugs >= 3, 1, 0), levels=c(0,1), labels=c("<3","≥3")),
SevereADR = factor(ifelse(ADR_Severity=="Severe",1,0), levels=c(0,1)) ) # 3)
Logistic■■ ---------------------------------------------------------- covars <-
c("Age_Group","Sex","Drugs3","Hepatic_Renal_Dysfunction","BMI25","`Duration_≥14d`")
# ■■■ uni_list <- lapply(covars, function(v){ f <- as.formula(paste0("ADR ~ ", v))
m <- glm(f, data=df, family=binomial) broom::tidy(m, conf.int = TRUE, exponentiate =
TRUE) }) uni_tab <- bind_rows(uni_list, .id="Variable") # ■■■ multi_fml <-
as.formula("ADR ~ Age_Group + Sex + Drugs3 + Hepatic_Renal_Dysfunction + BMI25 +
`Duration_≥14d`") multi_fit <- glm(multi_fml, data=df, family=binomial) # 4) ROC■■
--------------------------------------------------------------- fit_multi <-
glm(SevereADR ~ Age_Group + Drugs3 + Hepatic_Renal_Dysfunction + `Duration_≥14d`,
data=df, family=binomial) roc_multi <- pROC::roc(df$SevereADR, fitted(fit_multi),
quiet=TRUE) png("Figure_ROC_AllModels.png", width=1400, height=1000, res=160)
plot(roc_multi, lwd=3, main="ROC: Severe ADR Prediction", legacy.axes=TRUE)
dev.off()
```
